# Is There Evidence to Support Probiotic Use for Healthy People?

**DOI:** 10.1016/j.advnut.2024.100265

**Published:** 2024-07-06

**Authors:** Daniel J Merenstein, Daniel J Tancredi, J Philip Karl, Alex H Krist, Irene Lenoir-Wijnkoop, Gregor Reid, Stefan Roos, Hania Szajewska, Mary Ellen Sanders

**Affiliations:** 1 Department of Family Medicine, Georgetown University Medical Center, Washington, DC, United States; 2 Department of Pediatrics, University of California, Davis, CA, United States; 3 Military Nutrition Division, United States Army Research Institute of Environmental Medicine, Natick, MA, United States; 4 Department of Family Medicine and Population Health, Virginia Commonwealth University, Richmond, VA, United States; 5 Department of Pharmaceutical Sciences, Utrecht University, Utrecht, The Netherlands; 6 Departments of Microbiology and Immunology and Surgery, Western University, London, ON, Canada; 7 Department of Molecular Sciences, Swedish University of Agricultural Sciences, Uppsala BioCenter, Uppsala, Sweden; 8 Research & Development, BioGaia AB, Stockholm, Sweden; 9 Department of Paediatrics, Medical University of Warsaw, Warsaw, Poland; 10 International Scientific Association for Probiotics and Prebiotics, Centennial, CO, United States

**Keywords:** probiotic, healthy human, USPSTF, vaginal infections, urinary tract infections, respiratory tract infections, gastrointestinal infections, cardiovascular health, International Scientific Association for Probiotics and Prebiotics, ISAPP

## Abstract

Probiotics are typically marketed as foods and dietary supplements, categories for products intended to maintain health in generally healthy populations and which, unlike drugs, cannot claim to treat or cure disease. This review addresses the existing evidence that probiotics are beneficial to healthy people. Our approach was to perform a descriptive review of efficacy evidence that probiotics can prevent urinary, vaginal, gastrointestinal, and respiratory infections, and improve risk factors associated with cardiovascular health or reduce antibiotic use. Other endpoints such as mental, dental, or immune health were not specifically addressed. We concluded that there is sufficient evidence of efficacy and safety for clinicians and consumers to consider using specific probiotics for some indications – such as the use of probiotics to support gut function during antibiotic use or to reduce the risk of respiratory tract infections – for certain people. However, we did not find a sufficiently high level of evidence to support unconditional, population-wide recommendations for other preventive endpoints we reviewed for healthy people. Although evidence for some indications is suggestive of the preventive benefits of probiotics, additional research is needed.

**Statements of Significance**

Many reviews address the value of probiotics for specific patient groups, but the strength of efficacy evidence for probiotic use by the general population for the prevention of disease has not been robustly addressed. We descriptively reviewed a subset of researched health endpoints, applying the high grade of evidence required by the United States Preventive Services Task Force to a general recommendation for a healthy population, and concluded that currently available evidence is not sufficient to recommend that probiotics be routinely used for prevention by the general population.

## Introduction

Probiotics are live microorganisms that, when administered in adequate amounts, confer a health benefit on the host [1]. Probiotics are widely used by consumers, endorsed by several guidelines, and often recommended by clinicians [2]. Reviews have addressed evidence-based use of probiotics for a variety of conditions, including for healthy and patient populations. Randomized controlled trials (RCTs) have been conducted for a variety of outcomes, including necrotizing enterocolitis, pouchitis, irritable bowel syndrome, and others, and probiotic use in family practice has been reviewed [3]. Our interest, however, was in evidence for probiotics in the prevention of disease in the general, healthy population. Some evidence exists for certain probiotic strains or blends to prevent infections of the urinary, vaginal, gastrointestinal (GI), or respiratory tracts, to support cardiovascular health and to reduce antibiotic usage.

A common question asked about probiotics is, “Should everyone take a probiotic?” In fact, there are few recommendations for *any* intervention to be used by people who are free of any underlying disease. Such interventions must have sufficient evidence of benefit and relatively little to no harm. In raising this question for probiotics, we considered the approach of an organization tasked with evaluating preventive evidence: the United States Preventive Services Task Force (USPSTF). Because an important component of a USPSTF review is the potential for harm, it is important to note here that experts considering the safety of probiotics recently concluded that commonly used probiotic strains are safe for use in the general population [4].

The USPSTF makes evidence-based recommendations for clinical preventive services, including screenings, counseling, and preventive medications, most often for healthy people or subgroups of patients. Their reviews utilize an approach that is rigorous, systematic, objective, and evidence-based while evaluating benefits and harms. Recommendations address services offered in a primary care setting and are given grades of A, B, C, D, or I. If A or B grade is given, it is suggested that the service be provided. The D grade means the service should be discouraged, and an I grade means there is insufficient evidence to make a determination. The C grade means no recommendation for or against routine provision of the service because the benefits do not outweigh the potential harms [5].

The USPSTF recognizes that preventive measures are difficult to study. Healthy people recruited into a study are often unlikely to become unhealthy, especially over the short term. So studies must either be long-term or must identify more immediate endpoints, such as validated biomarkers or infectious disease, as targets for prevention. Furthermore, the threshold of evidence for recommending any intervention to a healthy population has to be very high, partially due to the potential risk of harm. In a patient with an illness, a risk of harm may be tolerable if the benefit outweighs risk. However, in an asymptomatic individual, this threshold is more difficult to determine. Some preventive measures are widely believed by the general public to be effective, but upon scrutiny of the data have been found to lack supporting evidence. For example, a systematic approach to evaluating evidence led the USPSTF to conclude that there is insufficient evidence to recommend a daily multivitamin for the prevention of cancer or cardiovascular disease (CVD) [6]. As one would expect for recommendations for healthy people, the USPSTF imposes a high bar for required evidence.

We assembled a group of experts to explore available evidence (studies summarized in Table 1) [[7], [8], [9], [10], [11], [12], [13], [14], [15], [16], [17], [18], [19], [20], [21], [22], [23], [24], [25], [26], [27], [28], [29], [30], [31], [32], [33], [34], [35], [36], [37], [38], [39], [40], [41], [42], [43]] using a similar framework of USPSTF’s approach to assessing outcomes in healthy people. We also discussed the research needed to close evidence gaps. Our primary focus was healthy people, but we also considered at-risk populations or population subgroups where recommendations may be justified. A previous review addressed whether healthy people should take a probiotic supplement, but the focus of that article was an improvement in specific bacteria in the gut microbiota, whereas we evaluated outcomes that are clinically relevant [44].

**TABLE 1:** Details of reviewed studies.

	Reference	Study design	Population studied	Sample size (test, I and placebo, P)	Outcomes examined	Method of probiotic delivery	Strain(s) and dose (CFU/d^1^)	Control	Results
a. Individual trial reports									
UTI	Reid et al. [7]	R, DB	Women with history of recurrent UTI	I = 25 P = 24	Incidence of recurrence at 12 mo	Vaginal 1×/wk for 12 mo	10^9^*Lacticaseibacillus rhamnosus* GR-1 + 10^9^*Limosilactobacillus fermentum* B-54	Skim milk prebiotic	Probiotic reduced recurrence of UTIs
UTI	Stapleton et al. [8]	R, DB, PC	Women with history of recurrent UTI	I = 48 P = 48	Reduction in UTI recurrence	Vaginal daily for 5 d, then 1 × wk for 10 wk	10^8^ CFU/mL^2^*Lactobacillus crispatus* CTV-05	Gelatin capsule	Probiotic reduced recurrence of UTIs
UTI	Baerheim et al. [9]	R, PC	Women with history of recurrent UTI	I = 25 P = 22	Reduction in UTI recurrence at 6 mo	Vaginal 2×/wk for 26 wk	7.5∼10^8^*L. rhamnosus*^3^	Solid semisynthetic glycerides (97.3%) and colloidal silica (2.7%)	Probiotic did not reduce the recurrence of UTIs
UTI	Kontiokari et al. [12]	R, open label	Women with history of UTI	I.1 = 50 I.2 = 50 P = 50	First recurrence of UTI	Oral daily for 6 mo	50 mL/d cranberry-lingonberry juice for 6 mo or 100 ml/d, 5 d/wk, 4 × 10^10^*L. rhamnosus* GG drink for 1 y	No intervention	Probiotic was less effective at reducing recurrence of UTI compared with cranberry juice
UTI	Beerepoot et al. [13]	R, DB noninferiority trial	Postmenopausal women with history of recurrent UTI	I = 125 P = 127	Mean number UTIs, proportion with ≥ 1 UTI during 12 mo, time to first UTI, and development of antibiotic resistance by *Escherichia coli*	Oral daily for 1 y	10^9^*L. rhamnosus* GR-1 + *L. reuteri* RC-14 2×/d	Trimethoprim Sulfamethoxazole, 480 mg, once daily	Probiotics were less effective at preventing UTIs than antibiotics, but do not increase antibiotic resistance
BV	Ya et al. [14]	R, DB, PC	Women with history of ≥2 BV episodes in the previous year	I = 58 P = 62	BV recurrence rate	Daily vaginal use 7 d on, 7 d off, and 7 d on	8 × 10^9^*L. rhamnosus*, *L. acidophilus* + *Streptococcus thermophilus*^2^	Capsules	Probiotics reduced recurrence of BV
BV	Mezzasalma et al. [15]	R, DB, 3-arm parallel pilot study	Healthy premenopausal women	I.1 = 20 I.2 = 20 P = 20	Detection of the strains in the vagina up to day 21	Oral for 14 d	2 preparations were tested. Each probiotic strain delivered at 10^9^: *L. acidophilus* PBS066*, L. reuteri* PBS072 + 320 mg inulin; or *L. plantarum* PBS067*, L. rhamnosus* PBS070*, Bifidobacterium animalis* subsp. *lactis PBS075 +* 298 mg inulin	Placebo containing 390 mg inulin	Probiotics were vaginally detected after 21 d
BV	Larsson et al. [16]	R, DB, PC	Women recently cured of BV	I = 50 P = 50	Time to relapse after cure	Vaginal gelatin capsules for 10 d during 3 menstrual cycles	10^9^ lactobacilli^3^	Matched placebo capsule	Probiotics decreased the recurrence of BV
BV	Bohbot et al. [17]	R, DB, prospective, multicenter, phase III	Women with history of documented recurrent episodes of BV	I = 39 P = 39	Rate of recurrence, time to recurrence	Vaginal capsules	10^9^ CFU/g^2^*L. crispatus* IP174178	Placebo capsule	Probiotics decreased the recurrence of BV and increased the time to recurrence
Reduction of antibiotic use	Ahrén et al. [18]	R, DB, PC	Healthy adults (18-70 y) with ≥4 common colds within past 12 mo	I = 448 P = 450	Severity, incidence rate, and duration of common cold; medication use (including Abx)	Oral powder 1×/d for 12 mo	10^9^*L. plantarum* HEAL9, 10^9^ CFU/d and *L. paracasei* 8700:2	Matched placebo	Probiotic intervention did not reduce antibiotic use
Community-acquired colds	Ahrén et al. [18]	R, DB, PC	Healthy adults (18–70 y) with ≥4 common colds within past 12 mo	I = 448 P = 450	Number of days of a cold episode	Oral powder 1×/d for 12 wk	10^9^*L. plantarum* HEAL9 and 10^9^*L. paracasei* 8700:2	Matched placebo	Probiotics reduced the incidence of colds in adults prone to getting colds
Reduction of antibiotic use	Butler et al. [19]	R, DB, PC	Residential and nursing care home residents (≥65 y)	I = 155 P = 155	Total days of Abx administration for all-cause infections; infections; AAD; health-related quality of life; hospital stays; deaths; *Clostridioides difficile* infection	Oral (capsule) for ≤1 y	1.3–1.6 x 10^10^*L. rhamnos*us GG + *B. lactis* BB-12	Matched placebo	Probiotics did not significantly reduce antibiotic administration for infections
CVD	Wastyk et al. [20]	R, DB, PC	Adults with elevated parameters of metabolic syndrome	I = 26 P = 13	Change in metabolic syndrome parameters	Capsule Daily for 10 wk	2 x 10^9^*L. reuteri* CIMB 30242, *L. plantarum* UALp-05 + *B. lactis* B420	Placebo	Dietary intake influences response to probiotics
Common GI and respiratory illness	Hatakka et al. [21]	R, DB, PC	Healthy children aged 1–6 y in daycare centers	I = 571 P = 289	Days with respiratory and GI symptoms; absences because of illness; number of URTIs; antibiotic treatments	Milk with or without *L. rhamnosus* GG	5–10 x 10^5^/mL *L. rhamnosus* GG in milk, 3×/d (compliance defined as ≥200 mL milk consumed/d), 5 d/wk, for 7 mo	Placebo milk	Probiotics may reduce GIs and RTIs in children
Common GI and respiratory illness	Hojsak et al. [22]	R, DB, PC	Children aged 1–7 y attending daycare centers	I = 139 P = 142	(1) Number of children with GI infections, defined as diarrhea with 3 or more loose or watery stools within 24 h with or without vomiting; (2) number of children with respiratory tract infections	Fermented milk product with or without live *L. rhamnosus* GG	10^9^*L. rhamnosus* GG for 3 mo	Placebo-pasteurized fermented milk	Probiotics decreased risk of upper RTIs in children

	Reference	Number of studies included	Study design(s) included in review	Population studied	Outcomes examined	Method of probiotic delivered	Strain(s) and dose	Control(s)	Quantitative results
b. Systematic reviews and meta-analyses									
UTI	Grin et al. [23]	*N* = 5	RCT	Premenopausal adult females	Incidence of recurrent UTIs		*Lactobacillus* spp.	Placebo	Probiotics reduce recurrent UTIs [pooled risk ratio of 0.51 (95% CI: 0.26, 0.99, *P* = 0.05)]
UTI	Ng et al. [24]	*N* = 9	Published clinical studies	Women with recurrent UTIs	Prophylactic efficacy and safety/incidence of adverse effects	Any	*Lactobacillus* spp.	Qualitative synthesis included 2 open, uncontrolled trials, 1 historic control. Studies in meta-analysis included only placebo-controlled trials	Probiotics reduce 1 recurrent UTI episode during the study [pooled risk ratio of 0.684 (95% CI: 0.438, 0.929; *P* < 0.001)]
RTI	Wang et al. [25]	*N* = 23	RCT	Infants, children and adolescents (birth to 18 y)	Number with ≥1RTI; RTI episode duration; total days of RTI; absenteeism; adverse events	Any	Any strain or strain combination at any dose	Placebo	Probiotics decrease number of subjects with ≥1 RTI episode [RR = 0.89, 95% CI: 0.82, 0.96, *P* = 0.004]. In children, probiotic reduced days of RTIs/person compared with placebo (MD = –0.16, 95% CI: 0.29, 0.02, *P* = 0.03), and had fewer days of absence from daycare/school (MD = –0.94, 95% CI: –1.72, –0.15, *P* = 0.02).
RTI	Coleman et al. [26]	*N* = 42	RCT	Adults (18–65 y)	Number with ≥ 1 RTI; RTI episode duration; total days of RTI; RTI symptom severity	Oral	Any strain or strain combination at any dose	Placebo	Probiotics reduced the risk of experiencing ≥1 RTI (RR = 0.91; 95% CI: 0.84, 0.98; *P* = 0.01), and total days (rate ratio = 0.77; 95% CI: 0.71, 0.83; *P* < 0.001), duration (Hedges’ *g* = −0.23; 95% CI: –0.39, –0.08; *P* = 0.004), and severity (Hedges’ *g* = –0.16; 95% CI: –0.29, –0.03; *P* = 0.02) of RTIs.
RTI	Li et al. [27]	*N* = 6	RCT	Adults (18–65 y)	Number with ≥1 RTI; total number of URTI; URTI episode duration; adverse events	Oral	Any strain or strain combination at any dose	Placebo	Probiotics compared with placebo reduced the incidence of URTIs (RR = 0.77; 95% CI: 0.68, 0.87; *P* < 0.0001; *I*^2^ = 26%), the episode rate of URTIs (rate ratio: 0.72; 95% CI: 0.60, 0.86; *P* = 0.0002; *I*^2^ = 99%), and the mean duration of 1 episode of URTI (MD = –2.66; 95% CI: –4.79, –0.54; *P* = 0.01; *I*^2^ = 80%).
RTI	Amaral et al. [28]	*N* = 21	RCT	Infants, children and adolescents (birth–18 y)	RTI incidence rate; adverse events	Oral	Any strain or strain combination at any dose	Placebo	*L. rhamnosus* LCA reduced the rate of RTIs compared with placebo (RR = 0.38; Crl 0.19-0.45).
RTI	Rashidi et al. [29]	*N* = 22	RCT	Any age	RTI incidence; URTI incidence; lower RTI incidence	Fermented dairy product	Any strain or strain combination at any dose	Not specified	Probiotic fermented dairy products, compared with placebo, protected against RTIs overall (RR = 0.81, 95% CI: 0.74, 0.89), in children (RR = 0.82, 95% CI: 0.73, 0.93), in adults (RR = 0.81, 95% CI: 0.66, 1.00), and the elderly population (RR = 0.78, 95% CI: 0.61, 0.98).
RTI and Abx	Zhao et al. [30]	*N* = 23	RCT	All ages	Number with ≥1 URTI and ≥3 URTI; URTI incidence rate; URTI episode duration; absenteeism; adverse events; antibiotic prescriptions	Any	Any strain or strain combination at any dose	Placebo or no treatment	Probiotics may reduce the number of participants with ≥1 URTIs (RR = 0.76; 95% CI: 0.67, 0.87; *P* < 0.001); likely reduce the number of participants with ≥3 URTIs (RR = 0.59, 95% CI: 0.38, 0.91; *P* = 0.02); may reduce the incidence rate of URTIs (rate ratio 0.82, 95% CI: 0.73, 0.92, *P* = 0.001); may reduce the mean duration of an episode of acute URTIs (MD = –1.22 d; 95% CI: –2.12, –0.33; *P* = 0.007); likely reduce the number of participants who used prescribed antibiotics for acute URTIs (RR = 0.58, 95% CI: 0.42, 0.81; *P* = 0.001).
RTI and Abx	Laursen et al. [31]	*N* = 12	RCT	Children attending daycare (3 mo to 7 y)	Number with ≥1 RTI; number with ≥1 URTI; number with ≥1 AOM; antibiotic use; absenteeism	Any	Any strain or strain combination at any dose	Placebo	Compared with placebo, *L. rhamnosus* GG reduced the duration of RTIs (MD = – 0.78 d, 95% CI: −1.46, −0.09). *B. lactis* BB-12 did not impact the duration of RTIs or absence from daycare.
AAD	Guo Q et al. [32]	*N* = 33	RCT	Children (0–18 y) receiving antibiotics	Incidence of diarrhea using the primary investigators’ definition (i.e., frequency, consistency of bowel movements)	Oral	Any strain or combination at any dose	Placebo, active alternative prophylaxis, or no treatment	Probiotics reduced the incidence of AAD from 19% to 8% compared with control (RR = 0.45, 95% CI: 0.36, 0.56; *I*^2^ = 57%, 95%).
AAD	Goodman et al. [33]	*N* = 42	RCT	Adults receiving antibiotics	Incidence of AAD	Oral	Any strain or combination at any dose	Probiotic intervention; a placebo, alternative dose, alternative probiotic strain, or no treatment control	Consumption of probiotics with antibiotics compared with control reduces the risk of AAD in adults by 37% (RR = 0.63, 95% CI: 0.54, 0.73, *P* < 0.00001)
AAD	Zhang et al. [34]	*N* = 8	RCT	Elderly adults (>65 y) receiving antibiotic	Incidence of AAD	Oral	Any strain or combination at any dose	Placebo	6 studies suggested probiotics could prevent AAD if used within 48 h of starting antibiotics (RR = 0.71, 95% CI: 0.71, 1.00, *P* = 0.05, *I*^2^ = 49%).
AAD	Szajewska and Kołodziej [35]	*N* = 21	RCT	Children and adults receiving antibiotics	AAD/diarrhea	Oral	*Saccharomyces boulardii*^4^ only	Placebo or no treatment	*S. boulardii* reduced the risk of AAD in children from 20.9% to 8.8% (RR = 0.43, 95% CI: 0.3, 0.6); in adults, from 17.4% to 8.2% (RR = 0.49, 95% CI: 0.38, 0.63); and reduced the risk of *C. difficile*-associated diarrhea only in children (RR = 0.25; 95% CI: 0.08, 0.73).
AAD	Szajewska and Kołodziej [36]	*N* = 12	RCT	Children and adults receiving antibiotics	AAD/diarrhea	Oral	*L. rhamnosus* GG only	Placebo or no treatment	In antibiotic-treated patients, *L. rhamnosus* GG compared with placebo or no treatment reduced the risk of AAD from 22.4% to 12.3% (RR = 0.49, 95%, CI: 0.29, 0.83).
Travelers’ diarrhea	McFarland and Goh [37]	*N* = 12	RCT	Children or adults	incidence of TD	Oral	≥2 RCTs with the same probiotic strain or mixture	Placebo or no treatment	*S. boulardii* CNCM I-745, but not *L. rhamnosus* GG or *L. acidophilus*, reduced TD incidence compared with control (RR = 0.79, 95% CI: 0.72, 0.87; *P* <0.001).
Abx use	King et al. [38]	*N* = 17	RCT	Infants and children	Percent prescribed Abx; number of Abx Rx; days of Abx use	Oral	Any strain or strain combination at any dose	Placebo or no treatment	Placebo-treated, infants and children administered probiotics to prevent acute illnesses had a reduced risk of being prescribed antibiotics (RR = 0.71, 95% CI: 0.54, 0.94).
Abx use	Scott et al. [39]	*N* = 16	RCT	Infants and children (birth to 18 y)	Abx use; number with ≥1 AOM; AOM severity; adverse events; AOM episode duration; absenteeism	Any	Any strain or strain combination at any dose	Placebo, usual care, or no probiotic	Probiotics reduced the proportion of children experiencing ≥1 episodes of AOM (RR 0.77, 95% CI: 0.63, 0.93)
CVD	Dong et al. [40]	*N* = 18	RCT	Children and adults	Metabolic syndrome parameters	Oral intake of probiotic food or supplement	Any strain, form, dose, and duration	Placebo	There were no significant differences between intervention and control groups in numerous anthropomorphic and biochemical outcomes, except standardized mean net differences in the body fat percentage (95% CI: –0.64, 0.03, *Z* = 1.81, *P* = 0.07) and LDL-C (95% CI: –0.34, –0.03, *Z* = 2.36, *P* = 0.02 < 0.05).
CVD	Koutnikova et al. [41]	*N* = 105	RCT	Adults and children >3 y	Obesity, diabetes, and NAFLD variables	Oral	Any lactic acid bacterium or *Bifidobacterium*, any dose and ≥14 d duration	Placebo	In overweight but not obese subjects, probiotics induced improvements in body weight (d =–0.94 kg MD, 95% CI: –1.17, –0.70), body mass index (*d* = –0.55 kg/m^2^, 95% CI: –0.86, –0.23), waist circumference (*d* = –1.31 cm, 95% CI: –1.79, –0.83), body fat mass (*d* = –0.96 kg, 95% CI: –1.21, –0.71) and visceral adipose tissue mass (d= –6.30 cm^2^, 95% CI: –9.05, –3.56). In type 2 diabetics, probiotics reduced fasting glucose (d = –0.66 mmol/L, 95% CI: –1.00, –0.31), glycated hemoglobin (d = –0.28 pp, 95% CI –0.46 to –0.11), insulin (*d* = –1.66 mU/L, 95% CI: –2.70, –0.61) and homeostatic model of insulin resistance (d = –1.05 pp, 95% CI: –1.48, –0.61).
CVD	Arabi et al. [42]	*N* = 5	RCT	Adults	Metabolic syndrome parameters	Oral	Any synbiotic product		Synbiotic intervention significantly reduced serum insulin levels (WMD, –6.39 μU/mL, 95% CI: –7.2, –5.4, *P* = 0.001), triglycerides (WMD, –20.3 mg/dL, 95% CI: –32.7, –7.8, *P* = 0.001), total cholesterol (WMD, –7.8 mg/dL, 95% CI: –12.5, –3.02, *P* = 0.001), low-density lipoprotein cholesterol (WMD, –9.02 mg/dL, 95% CI: –10.8, –7.2, *P* < 0.001), waist circumference (WMD, –4.04 cm, 95% CI: –4.9, –3.08, *P* < 0.001), body weight (WMD, –4.3 kg, 95% CI; –6.2, –2.5), systolic blood pressure (WMD, –1.8 mmHg, 95% CI: –2.8, –0.7, *P* = 0.001), and serum interleukin-6 concentrations (WMD, –0.2 pg/mL, 95% CI: –0.3, –0.08, *P* = 0.001), and increased high-density lipoprotein cholesterol levels (WMD, 2.3 mg/dL, 95% CI: 0.2, 4.4, *P* = 0.03).
CVD	Hadi et al. [43]	*N* = 10	RCT	Adults	Metabolic syndrome parameters	Oral	Any probiotic or synbiotic	Placebo	Probiotics/synbiotics reduced total cholesterol in adults with metabolic syndrome compared with placebo (MD = –6.66 mg/dL, 95% CI: –13.25, –0.07, *P* = 0.04)

Abbreviations: Abx, antibiotic; AOM, acute otitis media; BV, bacterial vaginosis; CI, confidence interval; CrI, credible interval; DB, double-blind; GI, gastrointestinal infection; MD, mean difference; NAFLD, nonalcoholic fatty liver disease; PC, placebo-controlled; R, randomized; RR, relative risk; RTI, respiratory tract infection; Rx, prescription; URTI, upper RTI; WMD, weighted mean difference.

1 Unless otherwise indicated.

2 Daily dose was unclear from the article.

3 Paper does not provide strain designations.

4 The most commonly used *S. boulardii* strain in the included studies was *S. boulardii* CNCM I-745.

## Methods

The meeting to discuss this topic was convened under the auspices of the International Scientific Association for Probiotics and Prebiotics (ISAPP). ISAPP is a nonprofit organization dedicated to advancing the science of probiotics, prebiotics, and related substances. ISAPP is funded by membership dues from companies and nonprofit organizations that share ISAPP’s mission, but ISAPP activities are determined by an all-volunteer, academic voting board of directors. ISAPP provides travel funding for experts to attend annual meetings and participate in small groups to discuss compelling topics in the field. These groups are composed of invited academic experts and scientists from member companies. The discussion group participants include this article’s authors and others listed in the acknowledgment section. The group undertook a nonsystematic approach to review available evidence for any probiotics (all strains and strain combinations) as an initial step to inform whether and which probiotic/outcome combinations were such that they could be nominated to the USPSTF as worthwhile candidates to undergo their more stringent and systematic review process. Experts chose specific endpoints to assess and each expert collated evidence as he/she deemed appropriate. Concluding evidence statements were considered for probiotics as a category, although strain designations for all included probiotics were provided.

Our goal was to focus on a few endpoints where probiotics have been assessed for prevention. We largely relied on systematic reviews conducted by other groups. We focused on evidence that any probiotic preparation could prevent urinary tract infections (UTIs), prevent vaginal tract infections, prevent GI tract infections, prevent respiratory tract infections (RTIs), improve risk factors associated with cardiovascular health, or reduce antibiotic use.

We made a preliminary judgment that these endpoints were the most likely to provide adequately robust information to lead to a general conclusion about the usefulness of certain probiotics for prevention. Although probiotics have been studied in healthy or at-risk populations for other endpoints including dental health, mental health, and prevention of allergy, we did not consider it likely that evidence was sufficient in these more nascent fields of investigation to warrant their consideration herein.

### Description of evidence for specific indications

#### Prevention of urinary tract infections in females

Symptomatic, culture-positive UTIs are the most common bacterial infection in the world, accounting for ∼25% of all infections in females [23]. Over 500,000 females in Canada require antibiotic treatment for UTIs by the age of 24 [45]. To treat recurrent infections, low-dose antibiotics are often prescribed for 12 mo to kill pathogens entering the bladder and stopping the cycle of infections [46]. Other options include self-administered short courses of antibiotics upon symptom development, daily or twice daily vaginal rinses with methenamine hippurate (100 mg) or povidone-iodine (for catheter use) to stop pathogen ascension into the bladder, although consumption of cranberry juice, antioxidants, garlic, and Echinacea are unproven [[46], [47], [48]].

The rationale for probiotic lactobacilli to prevent UTIs is that they inhibit the uropathogens that ascend from the rectum to the perineum, vagina, and urethra into the bladder. In females who have never experienced a UTI, these anatomical areas are typically colonized predominantly by lactobacilli [49]. Thus, boosting the abundance of lactobacilli in females where these organisms are depleted or not sufficiently protective against uropathogens is a potential means to prevent infection. Strains of potential interest to reduce risk of infection in otherwise healthy females have been tested in preclinical studies for characteristics including immunomodulatory properties and uropathogen inhibition, and production of antimicrobial substances such as hydrogen peroxide [50,51].

The modes of administration that have been tested for probiotics to prevent UTIs are either oral or direct installation into the vagina and around the perineum. Oral administration has been shown to result in the probiotic reaching the urogenital site, albeit in low numbers [52]. An advantage of the oral approach is that products can be developed as dietary or nutritional supplements and they can potentially inhibit ascension of the uropathogens from the intestine. The intravaginal application encompasses a drug or cosmetic regulatory pathway, which entails higher costs for development, but this approach results in higher counts of the probiotic to the site.

The literature on probiotics to support urogenital health in females comprises only a few trials via either the oral or vaginal routes. The strongest efficacy data as measured by systematic review and meta-analyses comes from studies in adults; insufficient evidence exists for any strains in children [23,24,53].

In a randomized, double-blind trial, weekly vaginal application for 12 mo of suppositories containing 10^9^ *Lactocaseibacillus rhamnosus* GR-1 with 10^9^ *Limosilactobacillus fermentum* B-54 lowered the UTI recurrence rate in 17 subjects compared with the previous 12 mo from 6 to 1.2 per annum; 21 females treated with a prebiotic delivered in skim milk had a similar result [7]. In a randomized, placebo-controlled study of young females with a history of recurrent UTI, antimicrobials were administered for acute UTI and then subjects were randomly assigned to receive either *Lactobacillus crispatus* CTV-05 or placebo daily for 5 d, then once weekly for 10 wk. The recurrent UTI episodes were reduced by the probiotic compared with placebo (7 of 48 compared with 13 of 48) [relative risk (RR), 0.5; 95% confidence interval (CI): 0.2, 1.2] [8]. In another study, 47 females with a history of 3 or more UTI episodes in the previous 12 mo were randomly assigned to receive 2.8 g vaginal suppositories containing either 7.5 x 10^8^ *L. rhamnosus* or placebo [solid semisynthetic glycerides (97.3%) and colloidal silica (2.7%)] twice weekly for 26 wk [9]. The resultant UTI rate at 6 mo did not differ between the groups (1.41) (95% CI: 0.88, 1.98).

Three studies examined the potential use of the probiotic, *L. rhamnosus* GG, for urogenital health. In one open-label trial, 42 postmenopausal healthy females consumed yogurt containing 1–2 × 10^9^ CFU/d of the strain for 1 mo. The probiotic was recovered in the vaginas of only 4 females (9.5%) despite being present in the stool of 33 females (78.6%) [10].

In another study, 10 healthy females vaginally inserted a capsule containing either *L. rhamnosus* GR-1 plus *Limosilactobacillus reuteri* RC-14 or *L. rhamnosus* GG for 3 consecutive nights. Vaginal swabs were taken before and at various time points after probiotic use. Strains GR-1 and/or RC-14 persisted in the vagina for ≤19 d, whereas *L. rhamnosus* GG was detectable ≤5 d [11]. One open randomized controlled trial of *L. rhamnosus* GG on UTI prevention was conducted in 50 females consuming a beverage containing 4 × 10^10^ *L. rhamnosus* GG/100 mL 5 d/wk for 1 y. No reduction in UTI incidence was observed (43% compared with 30% with placebo) [12]. At 6 mo, 19 (39%) subjects in the *L. rhamnosus* GG group, and 18 (36%) in the control had suffered ≥1 recurrence. Taken together, these results suggest that GG is not effective at preventing UTIs.

With oral administration, a combination of *L. rhamnosus* GR-1 and *L. reuteri* RC-14 has been shown to reduce the recurrence of UTI compared with the common antibiotic treatment, trimethoprim-sulfamethoxazole treatment. Beerepoot et al. [13] conducted a double-blind, randomized noninferiority trial of 252 postmenopausal females with recurrent UTIs. Subjects were randomly assigned to 1 of 2 groups: 480 g of trimethoprim-sulfamethoxazole plus 1 placebo capsule twice daily or 1 capsule containing ≥10^9^ CFU each of *L. rhamnosus* GR-1 and *L. reuteri* RC-14 twice daily. Over the 12-mo study, the UTI rate for the probiotic group was reduced compared with baseline from 6.8 to 3.3 per year, whereas the antibiotic treatment reduced UTIs from 7.0 to 2.9 per year. The between-treatment difference of 0.4 UTIs per year (95% CI: −0.4, 1.5) was outside the noninferiority margin of 10%, indicating equivalency of the interventions [13]. The subjects provided samples of urine, feces, and a vaginal swab before the study medication was given, and each month until 3 mo after discontinuation of the medication. Drug-resistant *Escherichia coli* was isolated from 80% to 95% of subjects in the placebo group after 1 mo of antibiotic use. In contrast, the level in the probiotic group was unchanged at around 20% [54], providing an additional rationale for the use of probiotics instead of antibiotics in this scenario.

#### Prevention of vaginal tract infections

Infections of the vaginal tract are extremely common, caused by bacteria, yeast, and viruses. Particular concern arises with respect to reproduction, where the ability to conceive and take a fetus to term is adversely affected by a microbiota disrupted by pathogens [55,56]. Vaginal tract infections are characterized by a disruption of the normal microbiota. The vaginal microbiota of healthy females primarily contains species under the umbrella of “lactobacilli,” although this is not universally the case. About 25% of healthy females have a microbiota that is not dominated by lactobacilli microbiota but instead by facultative and obligate anaerobes [57,58].

Bacterial vaginosis (BV) results from an imbalance of bacteria that normally reside in the vagina, allowing pathogenic or opportunistic pathogenic bacteria, typically Gram-negative microbes such as *Gardnerella vaginalis*, to overgrow. Symptoms include discharge, vaginal fluid pH >4.5, fishy odor, and discomfort. There are both microbiological and symptomatic components to diagnosing BV, but vaginal dysbiosis can be asymptomatic [59,60]. Vaginal yeast infections (also called vulvovaginal candidiasis because the fungus *Candida albicans* is a causative agent) are also common, causing local irritation, vaginal discharge, and itchiness. Probiotics have been investigated for their effectiveness to prevent both BV and vaginal yeast infections.

The rationale for using probiotics to prevent these conditions is based on promoting a microbiota that resists the overgrowth of opportunistic pathogens. Probiotics have been administered with the goal of replenishing the vaginal microbiota with lactobacilli able to inhibit the growth of the pathogens, interfere with pathogen adherence and biofilm formation on the epithelium, and promote the return of the host’s naturally occurring lactobacilli strains. Probiotics have been administered both orally and intravaginally in studies investigating efficacy. The latter has the advantage of delivering higher numbers of probiotic directly to the vaginal tract, which may improve efficacy.

Studies conducted with probiotics to prevent BV are limited. Preclinical human studies documented the ability of orally administered probiotic strains to reach the vaginal tract [15,52]. Clinical outcomes were measured in a randomized, double-blind, placebo-controlled study in females with a history of ≥2 BV episodes in the previous year. Subjects were asked to report their occurrences of BV and if reporting symptoms of malodor or thin discharge, the diagnosis was confirmed by Amsel criteria at a clinic. The intervention in this study was intravaginal administration of a capsule containing 8 x 10^9^ CFU of *L. rhamnosus, Lactobacillus acidophilus,* and *Streptococcus thermophilus* (strains not reported) [14]. The subjects were randomly assigned to use 1 capsule/d of the probiotic (*n* = 58 females) or placebo (*n* = 62 females) for 7 d, followed by cessation of product for 7 d, and then restarted usage for 7 d. The females returned at 30 and 60 d after the second 7-day treatment to assess the vaginal microbiology. At a further 10 mo telephone follow-up, subjects were asked to report BV symptoms, diagnosis of BV or *G. vaginalis*, and adverse events over a 2–11-mo period. The study reported that the probiotic intervention resulted in lower recurrence rates of BV [15.8% (9 of 57 females) compared with 45.0% (27 of 60 females); *P* < 0.001] and *G. vaginalis* incidence through 2 mo [3.5% (2 of 57 females) compared with 18.3% (11 of 60 females); *P* = 0.02].

A multicenter, double-blind, randomized, placebo-controlled trial was conducted in females presenting with confirmed BV and also with a history of recurrent BV (≥2 documented episodes of BV in the previous year) [17]. All subjects were treated with metronidazole for 7 d before randomization. The study intervention was capsules of *L. crispatus* IP 174178 10^9^ CFU administered vaginally for 14–28 d over 112 d total. The primary endpoint was the prevention of bacteriologically confirmed and clinically diagnosed recurrence of BV at day 112. Other clinically relevant secondary endpoints were also tracked. A full analysis set of subjects (*n* = 98) included randomized subjects compliant with ≥1 treatment capsule; per-protocol subjects numbered 85. In both the full analysis set and per-protocol groups, probiotics reduced recurrence at day 112 by approximately half. Specifically, in the per protocol group, 16 of the 37 patients (43.2%) in the placebo group presented with ≥1 recurrence of BV (90% CI: 29.8, 56.6) compared with 8 of the 39 patients (20.5%) in the probiotic group (90% CI: 9.9, 31.1; *P* = 0.033).

Larsson et al. [16] conducted a double-blinded, randomized, placebo-controlled trial in females with clinically diagnosed BV. Although the primary endpoint of this study was therapeutic, and therefore out of scope for this article, the secondary endpoint addressed increased time to relapse after clinical cure. The therapeutic stage of this trial enrolled 100 females, and 76 of them who were cured of BV with clindamycin were carried forward to the next phase. The study duration was 6 menstrual cycles or until relapse during that time. The probiotic tested was intravaginally inserted capsules of *L. gasseri* Lba EB01-DSM 14869 combined with *L. rhamnosus* Lbp PB01-DSM 14870, with each strain present at a dose of ≥10^8–9^ CFU/capsule, 1 capsule/d. No impact on the initial cure rate was observed, but at the end of the study, the probiotic group had 64.9% BV-free subjects compared with the placebo group with 46.2%. Furthermore, the probiotic group had a greater time from cure to relapse than the placebo group (*P* = 0.027).

In conclusion, we considered studies that enrolled either healthy females or females with active cases of BV but were followed after cure for remission rates. Taken together, they total 4 RCTs. All studies investigated different probiotic preparations, with some studies using oral administration and others using intravaginal administration. All showed that the probiotic administered delayed recurrence. The involvement of dysbiotic microbiota in the etiology of BV allows a hypothetical mechanism between the live probiotic microbe and efficacy in promoting vaginal health. However, the data are limited.

#### Prevention of GI tract infections

*Prevention of antibiotic-associated diarrhea.* Much clinical research has focused on the use of probiotics to prevent antibiotic-associated diarrhea (AAD). Although antibiotics are used in unhealthy patient populations, they are also used prophylactically in a variety of situations, often to treat ambulatory conditions. Furthermore, probiotic use with antibiotics could in theory help sustain normal gut microbiota composition and function, in generally healthy people. Therefore, although the scope of this review is prevention in healthy populations, we opted to include data on probiotic use for AAD. AAD refers to diarrhea that emerges during or shortly after antibiotic use, with no other apparent cause [61]. Essentially, any antibiotic has the potential to induce AAD, which might appear hours after the initial dose or even several months after stopping the treatment. The likelihood of AAD depends on several factors, including its specific definition, the type of antibiotic prescribed, and patient factors. Particularly vulnerable are children younger than 6 mo, adults over 65 y, and hospitalized individuals [62]. In a recent systematic review of RCTs studying the impact of probiotics during antibiotic therapy, AAD was observed in 19% of pediatric control group participants, with reported rates varying from a low 2% to as high as 80% [32]. Among adults (≥65 y old), AAD occurrence ranges between 10% and 37% [62]. The symptoms of AAD can differ in severity, ranging from mild diarrhea to conditions such as colitis or pseudomembranous colitis [62]. Although *Clostridioides difficile* (previously *Clostridium difficile*) is the predominant infectious cause, especially in more severe AAD, other pathogens such as *Staphylococcus, Candida, Enterobacteriaceae,* and *Klebsiella* may be also involved [63].

Numerous systematic reviews, including a 2019 Cochrane review in children, found that most studied probiotics can reduce AAD risk [32]. A meta-analysis published in 2021, including 42 studies for a total of 11,305 subjects, reported that co-administration of probiotics with antibiotics reduces risk of AAD in adults by 37% [risk ratio (RR) = 0.63 (95% CI: 0.54, 0.73), *P* < 0.001] [33]. Using the grading of recommendations assessment, development and evaluation (GRADE) criteria, the overall quality of the evidence was found to be moderate. Design limitations of many of the included RCTs were balanced by a good magnitude of effect and dose–response gradient, thus increasing the certainty of the body of evidence.

A meta-analysis of 8 RCTs in 4691 elderly subjects reported a reduction in the incidence of AAD [34]. Although the authors recommend that elderly individuals be routinely distributed probiotics within 48 h of the first dose of antibiotics to prevent AAD development, this derives from only 5 RCTs. More large-scale RCT studies for the elderly are needed to confirm the preventive effects of probiotics in this age group.

Single-strain meta-analyses of RCTs found that compared with placebo or no intervention, probiotics such as *Saccharomyces boulardii* (the most commonly used strain investigated is CNCM I-745) or *L. rhamnosus* GG were most effective [32,35,36]. Given these findings, in 2023, the European Society for Pediatric Gastroenterology, Hepatology and Nutrition (ESPGHAN) recommended *L. rhamnosus* GG or *S. boulardii* for preventing AAD. For cases of *C. difficile*-related AAD, ESPGHAN conditionally recommended *S. boulardii* [64]. The World Gastroenterology Organization (WGO), for children, also recommends *S. boulardii* or *L. rhamnosus* GG*.* In addition, WGO recommends multispecies probiotic comprising *Bifidobacterium bifidum* W23, *Bifidobacterium animalis* subsp. *lactis* W51, *L. acidophilus* W37, *L acidophilus* W55, *Lacticaseibacillus paracasei* W20, *Lactiplantibacillus plantarum* W62, *L. rhamnosus* W71, *and Ligilactobacillus salivarius* W24; or *L. rhamnosus* (strains E/N, Oxy, and Pen). For adults, WGO offers a long list of probiotic options including yogurt with specific strains or multispecies probiotics. Unlike ESPGHAN, WGO bases its recommendations on just 1 RCT [2]. Meanwhile, in 2020, the American Gastroentereological Association chose not to review evidence for AAD prevention using probiotics [65]. Yet, they did suggest specific probiotics to reduce risk of *C. difficile* infections in antibiotic-treated children and adults.

In conclusion, the data for a moderate protective effect of probiotics in the prevention of AAD are overall supportive. A specific recommendation for the use of probiotics, in particular for vulnerable age groups, might be actively developed based on future well-designed studies on optimal timing, dose, and duration of probiotics more systematically associated with a prescription of antibiotics. Research to utilize probiotic strains mechanistically suited to be effective against AAD could improve the success of future human trials.

*Prevention of travelers’ diarrhea.* Travelers’ diarrhea (TD) is a common health concern for travelers. Although it can occur anywhere, risk is higher when traveling from developed to less-developed regions of the world [66]. A systematic review of RCTs from 2019 found that only 3 probiotics (*S. boulardii, L. rhamnosus* GG, and unspecified strains of *L. acidophilus*) were studied for preventing TD [37]. Among these, only *S. boulardii* demonstrated significant efficacy. Another systematic review of RCTs, with network meta-analysis, examined the comparative effectiveness of probiotics and rifaximin in the prevention of TD [67]. Both probiotics (RR: 0.85; 95% CI: 0.76, 0.95) and rifaximin (RR: 0.47; 95% CI: 0.35, 0.63) were associated with a significantly lower incidence of TD when compared with placebo, with rifaximin superior to probiotics in lowering TD incidence. The data supporting the use of probiotics for either prevention or treatment of TD were not sufficient to make a graded recommendation.

*Prevention of community-acquired diarrhea.* Children attending daycare centers face an increased risk of infections of the GI tract. Preventing these infections not only benefits the children and their families but also society. To address this, several probiotics have been studied for their potential in preventing community-acquired diarrhea. For instance, a study in Finland involving 571 children aged 1–6 y in daycare centers found no reduction in diarrhea but did report fewer absences due to GI and respiratory infections [21]. In addition, there was a reduced likelihood of antibiotics being prescribed for RTIs. However, the efficacy of *L. rhamnosus* GG is not universally supported. A randomized, double-blind controlled study by Hojsak et al. [22] found no significant differences in the occurrence or duration of GI infections among 281 Croatian children aged 13–86 mo who were administered *L. rhamnosus* in fermented milk.

The use of probiotic strains tested to date for preventing various types of GI illnesses, including AAD and community-acquired diarrhea, has shown mixed results. Although some strains such as *L. rhamnosus* GG and *S. boulardii* appear promising, especially for AAD, the evidence is not consistently strong across all conditions. Future studies should focus on establishing optimum dosages, durations, and strains to achieve the best clinical outcomes.

#### Prevention of RTIs

RTIs are a major worldwide public health threat. Illnesses resulting from RTI reduce the quality of life and productivity. They are a frequent reason for seeking outpatient medical care and for hospital admissions, and cause >2 million deaths annually [[68], [69], [70], [71]]. Low-cost, effective strategies for RTI prevention in healthy populations could, therefore, have widespread benefits at both the individual and population levels.

Mechanisms by which probiotics may prevent RTI are multifaceted. Plausible mechanisms include stimulating various components of both innate and adaptive immunity, strengthening epithelial barriers, producing antipathogenic compounds and directly interacting or competing with pathogens. These potential mechanisms are in some instances strain-specific but can also be shared across microorganisms of larger taxonomic groups such as genera or species, or even among categories such as Gram-positive or Gram-negative bacteria [1,72,73]. This suggests that probiotic interventions could plausibly impact RTI risk through shared mechanisms, but strain-specific and possibly pathogen-specific effects are also likely. Therefore, when evaluating whether sufficient evidence exists to support recommending probiotic interventions for RTI prevention in healthy populations, high-quality systematic reviews, and meta-analyses that group all probiotics into a single class of intervention or that consider genus-, species-, strain- or disease-specific effects can all inform our understanding of the evidence [72].

Evidence regarding the use of probiotics for RTI prevention is largely derived from clinical studies published within the past 2 decades reporting orally ingested interventions. Collectively, those studies have included thousands of generally healthy child and adult participants while testing various probiotic interventions, primarily using strains within the genera *Bifidobacterium, Lactobacillus, Lactocaseibacillus, Limosilactobacillus,* and *Lactoplantibacillus.* Recent meta-analyses of randomized controlled trials within that evidence base are largely consistent in reporting that probiotic interventions reduce risk of experiencing 1 or more RTIs, reduce the incidence rate of RTI, and reduce the total number of days with RTI [[25], [26], [27], [28], [29], [30], [31]]. Though effect sizes vary across meta-analyses, reductions in the RR of experiencing 1 or more RTIs ranged from 9% to 24% with 95% CIs spanning a negligible 2% to an appreciable 37% risk reduction [25,26,30,31]. Reported reductions in the rate ratio of illness episodes and total days of illness are larger and range from 18% to 31% with 95% CIs spanning an 8% to 46% rate reduction [[26], [27], [28], [29], [30]].

Despite meta-analyses consistently reporting favorable effects of probiotics for RTI prevention, the studies demonstrated considerable heterogeneity in results across individual trials. That heterogeneity either could not be fully explored due to insufficient data or, when explored, was generally not explained by subgroup analyses based on factors such as study quality, population age, type of RTI or composition, dose or duration of intervention, among others. An additional reason for unexplained heterogeneity may be strain specificity [1]. However, few meta-analyses have empirically examined this possibility. That is likely due, in part, to the fact that relatively few individual strains or strain combinations have been tested in enough studies within a target population or against the same outcome to conduct a meaningful meta-analysis. Those that have do not show a clear benefit for RTI prevention [31,74]. Additionally, concerns regarding risk of bias, including publication bias, have also been reported [26,30]. As a result, meta-analyses implementing the GRADE scoring system have considered the evidence supporting probiotic interventions for RTI prevention to be low quality [27,30,31].

#### Reduction of antibiotic use

There is considerable worldwide interest in reducing antibiotic use due to the growing global burden of antibiotic resistance [75]. The importance of this issue to public health led us to decide that this topic was in scope for this review. Notably, antibiotics are often prescribed unnecessarily, particularly for RTI and other common infectious diseases [76,77]. Healthcare visits in outpatient settings comprise the primary setting where most antibiotics are prescribed [78]. Implementing interventions that prevent or reduce the incidence, duration, or severity of common infections (of any etiology) may therefore reduce antibiotic use in generally healthy populations. For example, separate modeling studies have estimated that probiotic supplementation could prevent millions of antibiotic prescriptions annually based solely on the estimated potential benefits of probiotics for RTI prevention [[79], [80], [81]].

Several trials testing the effectiveness of various probiotic interventions for preventing common infections such as RTIs or GI infections have assessed and reported antibiotic use as a primary or secondary outcome. A 2018 meta-analysis of RCTs that included 17 of those trials reported that infants and children given probiotics to prevent common infections had a 29% (95% CI: 0.54, 0.94) lower RR of being prescribed antibiotics [38]. Heterogeneity was low after a single study was removed and the effect size was increased when only 5 trials considered to have a low risk of bias were included [RR = 0.46 (95% CI: 0.23, 0.97)] [38]. Other meta-analyses of RCTs have also reported that infants and children given probiotics to prevent RTI had a lower RR of being prescribed antibiotics, with effect sizes ranging from a 31% to 41% risk reduction and 95% CIs spanning a 5%–59% reduction in risk [30,31,39]. The overall quality of that evidence has been rated as low or moderate using GRADE scoring, with substantial heterogeneity and/or unclear risk of bias cited as reasons for uncertainty [30,31,38,39]. Notably, all trials included in those meta-analyses were conducted in infants and children. Two double-blind, randomized controlled studies conducted in adults that were not included in the meta-analyses failed to show that the probiotic interventions tested reduced antibiotic use [18,19]. Specifically, the combination of *L. plantarum* HEAL9 and *L. paracasei* 8700:2 when consumed daily for 1 y reduced the incidence of common colds but did not reduce antibiotic use (secondary outcome) relative to placebo in healthy adults with a history of frequent colds [18]. Likewise, consuming a combination of *L. rhamnosus* GG and *B. lactis* BB-12 (1.3–1.6 × 10^10^ cells/d) for ≤1 y did not reduce antibiotic administration (primary outcome) relative to placebo in care home residents aged >65 y [19].

Taken together, there does not appear to be strong evidence to support probiotic interventions for reducing antibiotic use in healthy adults at this time. In contrast, results from studies conducted in infants and children are promising. However, interpreting those results is challenging as antibiotic use has not been measured as a primary outcome. In addition, if any effects of probiotic interventions on risk or duration of infectious disease are strain-specific, impacts on antibiotic use may also be strain-specific. However, too few studies using the same intervention have been included in available meta-analyses to conduct a meaningful strain-specific analysis, and in 1 meta-analysis of 3 studies using *L. rhamnosus* GG, antibiotic use was not reduced [31]. Additional high-quality RCTs of probiotic intervention that define antibiotic use as a primary outcome, ideally, or as a secondary outcome are needed to help support future recommendations.

#### Maintain cardiovascular health or prevent CVD

CVD is a formidable global health challenge, claiming more lives than any other cause [82]. Maintaining cardiovascular health and preventing CVD is multifaceted, and the disease correlates with several lifestyle factors including diet, smoking, use of antibiotics, and early-life events such as mode of delivery, as well as risk factors such as hypertension, diabetes mellitus, and lipid disorders [[83], [84], [85], [86], [87]]. Several of those factors have a link to the gut microbiome and recent research has delved into the intricate relationship between the gut microbiome and cardiometabolic health [88]. The progression of cardiometabolic and CVDs, from a healthy state into a clinically silent CVD (including metabolic syndrome and insulin resistance) and finally into a clinically overt CVD highlights its strong connection to early alterations in the gut microbiota, including a reduction in microbiome richness, a decline in butyrate-producing bacteria, and an increase in pathobionts [89].

In line with the strong correlation between the above mentioned lifestyle factors, most of the efforts to prevent CVD are focused on dietary interventions, reduction of smoking, and medications to reduce blood pressure and improve blood lipids. However, the correlation between CVD and the microbiota composition and activity also increases the interest to explore the potential of probiotics, as well as fermented foods, in preventing or managing CVD. Due to the slow progression of CVD, no studies have been designed to explore the ability of probiotics to prevent disease, but some studies have evaluated probiotics in the reduction of risk factors such as hypertension, glucose metabolism, and obesity, among others [[90], [91], [92]].

Two systematic reviews of RCTs have evaluated the effects of probiotics on metabolic syndrome. Although the inclusion criteria differed substantially, both suggest that probiotics may have a favorable, albeit marginal impact on metabolic syndrome components. The first review included 18 studies with a range of outcome measurements. Both the study protocols and described outcomes showed substantial heterogeneity, and significant differences between the intervention and control groups were only reported for body fat percentage (standard mean difference: –0.30%) and low-density lipoprotein cholesterol (–0.16 mg/dL) [40]. The second review included 9 studies, also having substantial heterogeneity. Favorable effects on body mass index (variation from no difference to −1.0 kg/m^2^ in probiotics compared with control) and glucose metabolism (variation from no change to –7.5 mg/dL) were described, but despite those positive effects, the included studies were rated as nonrelevant for CVD prevention [83]. However, the authors also conclude that the effects probably are dose and strain-specific and that the duration of intervention may have been too short. A third systematic review with meta-analysis adopted a broad approach and investigated the impact of a range of probiotics on 15 variables related to obesity, diabetes, and nonalcoholic fatty liver disease [41]. Inclusion criteria were met by 105 articles covering a total of 6826 subjects. The authors conclude that the probiotic strains tested resulted in minor but consistent improvements in several metabolic risk factors in subjects with metabolic diseases. However, a high number of studies were conducted in only 1 country (Iran); when these were excluded, the sensitivity analysis revealed nonsignificant effect estimates for all parameters.

Although our scope focuses on probiotics, in the context of CVD, it is of interest to note that 2 reviews looked into the impact of complementary synbiotics (a combination of probiotics and prebiotics) on various markers of metabolic syndrome. A meta-analysis of 5 RCTs showed that the studied synbiotics significantly reduced serum insulin levels, triglycerides, total cholesterol, low-density lipoprotein cholesterol, waist circumference, body weight, systolic blood pressure, and serum IL-6 concentrations, and increased high-density lipoprotein cholesterol levels compared with placebo [42]. These results were both statistically significant and clinically relevant. Overall, the risk of bias and heterogeneity was low, but the small number of studies as well as the low sample size requires confirmation of the evidence from larger trials. Another analysis, based on ten eligible publications (9 RCTs, 344 participants) concluded that supplementation with probiotics or synbiotics compared with placebo reduced total cholesterol in adults with metabolic syndrome, but without affecting weight, body mass index, waist circumference, fasting blood sugar, homeostasis model assessment for insulin resistance, insulin, triglyceride, low-density lipoprotein cholesterol, or high-density lipoprotein cholesterol (*P* > 0.05) [43]. Most studies suffered from methodological limitations, differences in strains, dosage and duration of the interventions, and patient characteristics, which underlines again the need for more well-designed trials.

A recent randomized placebo-controlled trial using a mix of 2 strains of lactobacilli and 1 bifidobacteria on adults with metabolic syndrome indicated that the effect of the probiotic supplementation depended on the diet. A subset of the probiotic arm responded with improvements in triglycerides and diastolic blood pressure (*P* < 0.05) and those responders both had a distinct microbiome profile and higher intake of certain nutrients like sugars, calcium, zinc, and folate. This suggests that probiotics could be combined with a healthy diet to increase the efficacy and potentially reduce CVD risk [20].

## Discussion

Our aim of this article was to provide a descriptive evaluation of evidence that probiotics can prevent disease or maintain health, or in other words, an assessment of the value of probiotics for healthy people. Our conclusions for the endpoints discussed are that none of the efficacy data are yet sufficiently robust to meet the stringent criteria imposed by the USPSTF for a preventive recommendation. It is important to understand, however, that the conclusion that the data are not ready for a population-based USPSTF recommendation is not the same as saying the data are not sufficient to be considered for healthy people. Probiotic research is relatively new, and the absence of evidence of effectiveness is not the same as having evidence of ineffectiveness. In fact, we know that there are many specific circumstances where, considering available efficacy and safety evidence, probiotics are a prudent option. But the evidence is not yet sufficiently robust for recommendations for prevention in the general population.

We reviewed 6 prevention endpoints: UTIs and vaginal infections in women, RTI, GI conditions, CVD, and antibiotic use. For UTIs, we concluded that specific probiotic strains (*L. rhamnosus* GR-1 and *L. reuteri* RC-14) administered orally may help prevent UTIs in women, although the evidence for vaginally administered strains is lacking. We judged that the existing evidence would not be enough to request a USPSTF review. The evidence base for AAD and community-acquired diarrhea was the most robust of any outcome we examined and was supported by some independent guidelines. However, because of study heterogeneity and in some cases failure to identify the probiotic strains studied, the evidence is likely not sufficient for a recommendation by USPSTF. As was the case with other outcomes we evaluated, the biological plausibility was strong for probiotic prevention of RTIs and to decrease antibiotic usage. However, the evidence likely was still not robust enough for a recommendation if we apply USPSTF standards. With managing risk factors of CVD, probiotics likely have great promise and we are hopeful that future research will advance our understanding of the potential of biotics to contribute to cardiometabolic health. However, due to the complex, multifactorial causes of CVD and the limited number of studies, the data for a recommendation to use probiotic interventions to prevent CVD need to be further strengthened. As such, a parallel can be made with the USPSTF’s updated evidence review on the use of vitamin supplementation to prevent CVD and cancer, resulting overall in a D/I grade [93].

As mentioned, the bar for USPSTF needs to be extremely high for several reasons, most importantly because the recommendations are for healthy people. In this context, it is important to recognize that evidence for the safety of probiotics in the general population is strong [4]. In specific situations, such as preventing AAD in subjects receiving antibiotics, certain probiotics may be considered. If a patient is having recurrent UTIs or frequent RTIs, an evidence-based approach would entail talking with the patient about the evidence behind the strains we reviewed. In addition, any intervention needs to be weighed against its risk and costs. Many of the strains discussed herein have a strong record of safety and are relatively inexpensive. Further, aggregate data – which is considered here – may vary in its applicability to any individual. A healthy person may reasonably decide when considering his own need and individual response that probiotic use is warranted.

Although a current USPSTF analysis would probably result in an I grade for the use of probiotics in the prevention outcomes we assessed, it might only take a few well-designed studies to overcome the evidence gap, as illustrated by the cluster of consistent elements pointing to beneficial probiotic effects in prevention or risk reduction, and also supported by the currently available guidance issued by several specialized scientific societies and health organizations.

The most convincing evidence for the benefits of probiotics in disease prevention or risk reduction for a specific target population would come from 2 or more RCTs. These RCTs should be designed, conducted, and reported to minimize bias, involving well-characterized probiotics, appropriate dosages, and clearly defined outcomes. The evidence should be robust and precise enough to convincingly and directly demonstrate risk reductions for prespecified outcomes. Results should be generalizable to the target populations of interest [94]. In addition, the studies would characterize and report adverse effects, to permit a careful assessment of safety concerns.

When flaws in study design, conduct, and reporting are present, they necessitate that study results show higher probiotic benefit magnitudes to enable confidence that even when accounting for those flaws and the resulting potential mis-estimation of probiotic benefits and harms, one can be reasonably certain that the probiotic provides a net benefit in disease prevention or risk reduction outcomes for the target population and that those benefits convincingly outweigh potential safety concerns. Similarly, in cases where only a single study is available, one would expect that study to be of high quality and convincing and that there be a strong subject matter context for understanding how the intervention achieves its beneficial effects, to overcome potential generalizability concerns that arise from having to rely on only a single study that has not (yet) been corroborated.

The original aim of our discussion group was to assess evidence for the family of biotic substances (probiotics, prebiotics, synbiotics, and postbiotics) and fermented foods to prevent disease in healthy people [[95], [96], [97], [98]]. However, we focused on probiotics for this article in part because the evidence for probiotics was stronger than for the other substances and also for practical purposes to narrow the scope.

The evidence base for other biotic substances is expanding. For example, some postbiotics, defined as preparations of inanimate microorganisms and/or their components – with or without metabolic endproducts – that confer a health benefit, could potentially have a more potent impact on health parameters than the live microbe from which they were derived, as shown for a postbiotic preparation of a strain of *Akkermansia muciniphila*. In an exploratory study involving overweight and obese human volunteers, pasteurized *A. muciniphila*, though not live, demonstrated a favorable effect on several risk factors for CVD, including improved insulin sensitivity and reduced cholesterol. Although this study was exploratory, it hints at the potential of using specific postbiotic products to reduce the risk of CVD [99]. Furthermore, fermented food consumption, which may introduce a large number of live bacteria, dead bacteria, and bacterial components to the diet, has been associated with positive health outcomes. In a cohort study on >46,000 adults, using information from The National Health and Nutrition Examination Survey database, it was concluded that an additional 100-g intake of microbe-containing foods was associated with lower systolic blood pressure, C-reactive protein, plasma glucose, plasma insulin, triglyceride, waist circumference and BMI (kg/m^2^) levels, and a higher level of HDL cholesterols [100]. A similar effect was seen in a meta-analysis of cohort studies on the effect of yogurt consumption on the incident risk of CVD [101]. Those studies underscore the potential advantages of incorporating fermented foods into the diet for mitigating the progression of CVD.

We recognize that probiotics have been studied for endpoints on healthy subjects that we did not include. For example, prevention of allergy, cognitive function, mood, and prevention of dental caries have all been addressed in clinical trials. However, we judged that the evidence base for these was unlikely to be sufficient to meet the high bar required for a USPSTF recommendation. Therefore, we did not include these endpoints in our assessment. Our review did not consider the evidence for specific healthy subpopulations, such as athletes, pregnant women, healthy children, and the elderly [102]. We also did not consider probiotic use for the treatment of colic in infants, a generally healthy population. An individual patient data meta-analysis of double-blind, randomized placebo-controlled trials of high quality concluded that *L. reuteri* DSM17938 is effective in reducing crying time for breastfed infants with colic [103]. However, data for formula-fed infants with colic were lacking.

The USPSTF only has 5 recommended preventive medications – aspirin and statins to prevent CVD, medications to prevent breast cancer in high-risk women, folic acid to prevent neural tube defects, and ocular prophylaxis for gonococcal ophthalmia in newborns. For probiotics to make it onto this list, future probiotic studies would need to *1*) be high quality and designed to limit risks of bias – primarily double-blind randomized placebo-controlled trials, *2*) include healthy people or those potentially at risk for a condition that the probiotic would be preventing, *3*) demonstrate reduced risks for adverse health outcomes, consistent with the prevention, and *4*) rigorously monitor and report safety concerns. We hope this review will motivate researchers and funding sources to develop a path for needed research so the potential of probiotics to facilitate health can be realized.

## Acknowledgments

We thank Dr. Leila Shinn for taking comprehensive notes aiding the capture of key points of the discussion and Dr Jo-Ann Passmore for contributing to the discussion on the prevention of vaginal infections. We also thank all other members of the on-site discussion group, for their input into the discussion: Takuya Akiyama, Kirstie Canene-Adams, Andrea Doolan, Justine Fauvieau, Paul Gill, Justin Green, Erica Hill, Elizabeth McCartney, Kim Merselis, Junichi Minami, Frank Schuren, Seppo Salminen, Robert E. Steinert, Jessica van Harsselaar, Annemarieke van Opstal, and Elena Verdu. All authors attended the annual meeting of the International Scientific Association for Probiotics and Prebiotics (ISAPP) where this topic was developed. ISAPP paid travel expenses for all authors except SR, whose expenses were paid by BioGaia AB, and MES who did not have to travel to the meeting. Supporting sources had no involvement in or restrictions regarding publication.

## Author contributions

The authors’ responsibilities were as follows – DJM: conceptualized the project, developed the overall approach, oversaw the project, contributed written content, and had primary responsibility for final content; DJT: contributed to development of the overall project and approach, and contributed written content; JPK: had primary responsibility for contributing written content of a section of the article; AHK: contributed to development of the overall project and contributed written content; IL-W: contributed written content to the overall article; GR: had primary responsibility for contributing written content of a section of the article; SR had primary responsibility for contributing written content of a section of the article; HS: had primary responsibility for contributing written content of a section of the article; MES: contributed to development of the overall project and approach, contributed written content and coordinated the writing, assembly and editing of the article; and all authors: have read, edited as needed, and approved the final manuscript.

## Conflict of interest

The opinions or assertions contained herein are the private views of the authors and are not to be construed as official or reflecting the views of the United States Army or Department of Defense. Citations of commercial organizations and trade names in this report do not constitute an official Department of the Army endorsement or approval of the products or services of these organizations.

DJM has been an expert witness for VSL#3. He serves as a board member of the International Scientific Association for Probiotics and Prebiotics (unpaid, volunteer position). DJT is a member of the Scientific Advisory Council for Deerland Probiotics and has provided statistical consultations to Synbiotic Health and International Flavors and Fragrances. He serves as a board member of the International Scientific Association for Probiotics and Prebiotics (unpaid, volunteer position). JPK: No conflicts. AHK: No conflicts. IL-W: No conflicts. GR Consults for Seed, a company making pro/synbiotics. SR is employed part-time by BioGaia AB and is one of the co-founders of Ilya Pharma AB. HS has participated as a clinical investigator or advisory board member or consultant or speaker for: Arla, BioGaia, Biocodex, Danone, Dicofarm, Nestlé, Nestlé Nutrition Institute, Nutricia, Mead Johnson/RB, and Winclove. HS serves as a board member of the International Scientific Association for Probiotics and Prebiotics (unpaid, volunteer position). MES serves as consulting scientific advisor and formerly as executive science officer for the International Scientific Association for Probiotics and Prebiotics; she has consulted with Bayer, Pepsico, and the Bill and Melinda Gates Foundation; served on scientific advisory boards for Institute for Advancement of Food and Nutrition Sciences, United States Pharmacopeia, Danone NA, Sanofi and Cargill; and has been compensated for giving talks for Xpeer, Sanofi, European Federation of Association of Dietitians and Associated British Foods.

## Funding

ISAPP provided travel funding for DJM, DJT, AHK, IL-W, GR and HS to attend the meeting where this topic was discussed.
